# Principles in treating pediatric patients with pilonidal disease – An expert perspective

**DOI:** 10.1016/j.amsu.2021.102233

**Published:** 2021-03-27

**Authors:** Gregory A. Metzger, Jordan C. Apfeld, Leah Nishimura, Carley Lutz, Katherine J. Deans, Peter C. Minneci

**Affiliations:** a The Center for Surgical Outcomes Research, Nationwide Children's Hospital, Columbus, OH, USA; b The Department of Pediatric Surgery, Nationwide Children's Hospital, Columbus, OH, USA

**Keywords:** Pilonidal disease, Pediatrics, Trephination, Wound

## Abstract

Pilonidal disease is common amongst adolescent males and females and often leads to recurrent symptoms and life-altering morbidity. Traditionally, surgery included wide excision of the involved area with primary closure. Post-operative complication rates were high and recurrence of disease common, leading to a search for alternative approaches for treating pilonidal disease. A minimally invasive (trephination) approach was described by Gips in 2008 and has since been adopted by many surgeons. Although the trephination procedure is less morbid than total excision, the risk of wound complications is not insignificant and post-operative recurrence has been reported in more than 10% of patients. The lack of a clear advantage for any single treatment strategy has led a wide variation in provider approach. To standardize the care for pediatric patients with pilonidal disease, a dedicated clinic was created at our institution in 2018. The aim of this paper is to describe an approach to treating pediatric patients with pilonidal disease that has been established through the experience of treating hundreds of adolescent males and females per year. Given the impact on quality of life for those that are affected by pilonidal disease, it is important that future research be directed toward discovery of the best practices for treating this challenging disease.

## Highlights

• Pilonidal disease occurs frequently in adolescent males and females and leads to significant morbidity.
• Surgical options for patients with pilonidal disease frequently have post-operative complications and/or disease recurrence.
• Treatment strategies for pilonidal disease should be personalized and based on the limitations of the available techniques.
• Better treatment strategies for mitigating the negative effects of pilonidal disease on quality-of-life are needed for pediatric patients.

## 1 Introduction

Pilonidal disease is common amongst adolescent males and females, affecting approximately 26 in 100,000 individuals [1]. A complete understanding of the pathophysiology that underlies the disease remains to be found; however, several risk factors have been identified, including: a positive family history, the hirsutism, and poor hygiene [2,3]. Studies have failed to show a clear advantage for any single treatment strategy, leading to a heterogeneous assortment of treatment options and a wide variation in provider approach. Surgeons are often involved in the management of acute complications as well as chronic sequela, with the goal of treatment being to minimize recurrence rates and to mitigate the perceived disease burden. Unfortunately, the recurrence of symptoms after initial presentation is common, with up to 30% of patients experiencing multiple flares during the course of the disease [4]. Surgical options range from minimally invasive strategies to extensive flap procedures, but complete eradication cannot be guaranteed, and post-operative morbidity rates are high [5]. The purpose of this article is to share the principles of treating patients with pilonidal disease that have been established through experience treating patients at a dedicated pilonidal clinic. We established the pilonidal clinic at our institution in 2018 and currently evaluate approximately 12–15 patients with pilonidal disease per week. We remain dedicated to advancing the understanding of pilonidal disease through research and to improving the quality of life for our patients through personized treatment decisions.

## 2 Our approach

### 2.1 Basic principles

Studies exploring outcomes in pediatric patients with pilonidal disease have failed to show a clear advantage for any single treatment strategy [6,7]. As such, when counseling the patient with pilonidal disease it is important to consider certain patient-specific factors, including: 1) the chronicity of the presenting problem 2) the risk factors present 3) outcomes related to previous treatment attempts, and 4) the cumulative impact of the disease on the patient's quality of life. We aim to tailor treatment recommendations to the individual based on the expected disease course with consideration of patient preference following a thorough discussion of options and potential outcomes.

### 2.2 Initial evaluation

In most situations, imaging and labs are of limited utility when evaluating a patient with concern for pilonidal disease. We begin every encounter with an exam of the gluteal cleft, making note of the hair burden, the presence of any pits or wounds, and any signs of active infection. Alternative diagnoses to consider include hidradenitis suppurativa, infected furuncles, Crohn's disease/fistulas, and other infectious processes. It is important to palpate along the superior gluteal cleft and the coccyx to determine the relationship between elicited pain and skin changes in order to ascertain the location of underlying tracts and to exclude isolated bone pain as a potential diagnosis. It is essential to determine whether there are signs of active infection or abscess formation, as these conditions would need to be addressed prior to discussing long-term treatment options. An ultrasound can be considered if suspicion for pilonidal disease remains high despite physical exam findings that are discordant with the typical presentation. If the patient requires treatment for an acute flare, such as an incision and drainage to treat an abscess, then a follow up visit is scheduled 2–3 weeks after the procedure to confirm complete resolution of the infection before moving forward with discussion of long-term options. If the patient experiences a recurrent infection, then treatment is again aimed at resolving the issue, with drainage procedures or antibiotics being used as necessary. Given that no surgical option has been shown to reliably eradicate the possibility of recurrent pilonidal disease, we do not consider recurrence of an acute infection as an indication for more aggressive surgery. For patients electing for surgery at some point during the course of their treatment, we ensure that any acute infection has resolved prior to proceeding to the operating room. Any suspicion for a cancerous lesion will prompt a skin biopsy.

### 2.3 Preventative strategies (The conservative measures)

Pilonidal disease can affect patients of any age, but young adults constitute the group at greatest risk [8]. The pathophysiology surrounding pilonidal disease is not fully understood, but it is known that a positive family history and the presence of hair surrounding the gluteal cleft are both risk factors for disease development [9]. As such, we recommend that all patients adhere to three key principles regardless of treatment pathway. First, we ask that patients keep the gluteal cleft and the surrounding area free from hair [10]. This requires shaving the area every 2–3 weeks or as dictated by personal growth patterns. Chemical depilation can be considered in patients that do not have open wounds. Laser epilation is known to reduce the hair burden and can be considered; however, it is currently not covered by most insurance plans and out-of-pocket expenses may be prohibitive [11]. We provide patients and families with education on the expected area to keep hair free by performing the first shave in clinic and offering follow up instructions. The last two principles are related to the idea that pilonidal disease can be exacerbated by increased trauma and inflammation to the superior gluteal cleft [12]. The second principle includes minimizing pressure near the affected area. We educate patients on the importance of modifying sitting technique in order to avoid excessive pressure on the area. Given that our patient population is mostly school-aged, we have chosen to focus on techniques that can minimize impact without drawing the attention of peers, including placing pressure on the balls of the feet when sitting, leaning to one side, and crossing one's legs. The last principle is meant to minimize the risk of inflammation by decreasing the likelihood of hair and other debris perforating the skin. We advise that all patients avoid prolonged moisture near the affected area. This requires showering at least once per day and removing any sweaty garments as early as feasible following physical activity. Regardless of whether patients choose surgical or non-surgical strategies, we advise that these principles are followed until at least age 25 in order to minimize the chance of recurrence. The last preventive strategy that we address with all patients is recognition and early contact with our clinic. We do not routinely provide patients with prophylactic antibiotics. However, following the first flare most patients will be able to recognize pain in the affected area prior to the onset of physical signs. Therefore, to mitigate the effects of an acute flare and avoid prolonged discomfort, we ask patients to have a low threshold for contacting the clinic to arrange either a virtual or in-person visit to allow for earlier evaluation and potential initiation of antibiotics in patients reporting pain or skin changes.

### 2.4 Long-term management

The choice of long-term treatment modality is based on the patient's presenting condition, the perceived burden of disease, and results of previous treatment attempts. Pilonidal disease is a chronic condition with a high risk for recurrence and no surgical option has been shown to provide definitive treatment for all groups [7]. In addition, the risk of wound complication in patients choosing surgical intervention is not insignificant [4,5]. As such, we believe it is extremely important to counsel patients with these limitations in mind and to cater treatment decisions to the individual. We typically do not consider surgical intervention unless a patient has had multiple flares, has a painful cyst/nodule that would benefit from excision, or continues to be symptomatic, including having chronic fistula/wounds, despite use of preventative strategies. We do not use pre-defined, objective criteria as an indication for surgical intervention for patients with chronic wounds. Instead, we routinely engage patients and their families in an ongoing discussion of the risks and benefits of operative intervention in the context of their perceived burden of disease, taking into account their response to conservative therapy and the possibility of developing a post-operative wound complication. In select cases we will consider early surgical intervention in patients that present with their first flare but report a significant quality of life burden related to their disease or have chronic wounds. Patients that present with chronic wounds that choose to forgo surgical intervention require close monitoring, with appointments occurring every 1–3 weeks. To promote healing, silver nitrate is used in clinic when appropriate and patients are advised to perform daily salt-water baths. Chlorine baths can be substituted if the wound requires chemical debridement.

**Fig. 1:**
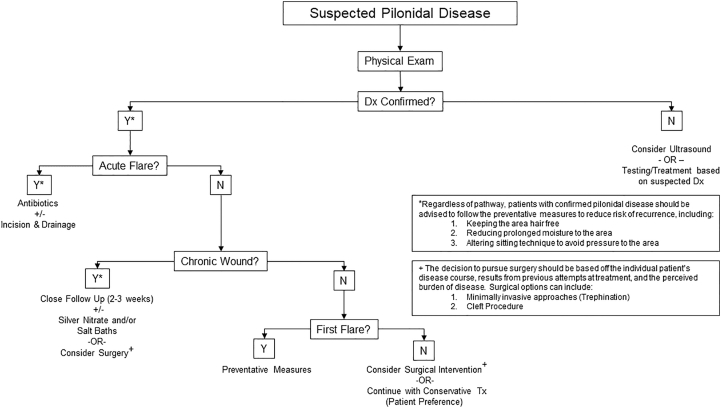
A personalized approach to treating the pediatric patient with pilonidal disease. Once a diagnosis is confirmed through physical exam, a treatment strategy is chosen that takes into account the patient's treatment history and perceived burden of disease. Regardless of the path chosen, all patients are encouraged to follow the preventative measures to minimize the chance for recurrence.

The traditional surgical approach to the patient with pilonidal disease included excision of the involved area down to the level of fascia with a midline re-approximation of the tissue. Studies have shown an increased morbidity associated with midline closure and a higher recurrence rate than off-midline closure [5]. As such, we no longer offer our patients excision with midline closure. There has also been a trend in recent years toward more minimally invasive surgical approaches, including trephination, and early reports indicate a reduction in the risk of disease recurrence comparable to more extensive surgery with fewer reported wound complications [13,14]. However, the results of this approach are in the early investigative phase. Given the lack of a proven surgical pathway, we offer patients with chronic or recurrent disease one of three options (Fig. 1):

1. No surgical intervention
2. Minimally invasive surgery (trephination)
3. Cleft procedure

We counsel patients on an individual basis, providing the risks and benefits of each of the above options and tailoring the treatment approach to the patient. For patients presenting to pilonidal clinic for the first time who are found to have signs of chronic disease, we advise a trial of non-operative management in an attempt to improve symptoms and minimize the burden of disease while avoiding the risks of surgery. Surgery is discussed with all families upfront, but it is typically only pursued if conservative management fails to adequately control the symptoms of disease, For patients interested in surgery, we counsel them on the risks and benefits of both the less invasive and more aggressive techniques. As with any surgery, there is the risk of anesthesia to consider. Studies of the more aggressive surgical approaches show a lower rate of disease recurrence than what has been reported with the trephination procedure, but they carry with them a higher rate of morbidity and are associated with a prolonged healing process and more frequent wound complications [[14], [15], [16]]. We will offer to repeat the trephination procedure for patients who experience a recurrence of their disease after surgery, and those electing to undergo the more aggressive surgeries are referred to plastic surgery, We discuss with patients that this is not a disease that will be cured through surgery alone and emphasize the importance of continuing the preventative measures regardless of pathway We make it explicit for all patients that this is a chronic disease and that any of the above treatment options will remain available throughout the course of treatment. .

## 3 Conclusion

Pilonidal disease is a common condition amongst adolescents that requires frequent intervention and often leads to life-altering morbidity. There is no single pathway that has been proven to consistently provide definitive treatment; providers must recognize the challenges associated with this disease and work with the patient to develop a treatment strategy that is best suited for the individual.

## Author contribution

Study conception and design: PM, KD, GM. Acquisition of data: GM, CL, LN, JA, PM. Analysis and Interpretation of data: GM, KD, PM. Drafting of Manuscript: GM, PM. Critical Revision: All authors.

## Financial disclosure

The authors have no financial relationships relevant to this article to disclose.

## Funding source

None.

## Provenance and peer review

Not commissioned, externally peer-reviewed.

## Guarantor

Peter Minneci, MD, MHSc.

## Declaration of competing interest

The authors have no conflicts of interest relevant to this article to disclose.
